# IMU-Based Fitness Activity Recognition Using CNNs for Time Series Classification

**DOI:** 10.3390/s24030742

**Published:** 2024-01-23

**Authors:** Philipp Niklas Müller, Alexander Josef Müller, Philipp Achenbach, Stefan Göbel

**Affiliations:** Serious Games Group, Technical University of Darmstadt, 64289 Darmstadt, Germany; alexander_j.mueller@outlook.com (A.J.M.); philipp.achenbach@tu-darmstadt.de (P.A.); stefan_peter.goebel@tu-darmstadt.de (S.G.)

**Keywords:** activity recognition, inertial measurement unit, deep learning, convolutional neural network, residual neural network, traditional machine learning, study

## Abstract

Mobile fitness applications provide the opportunity to show users real-time feedback on their current fitness activity. For such applications, it is essential to accurately track the user’s current fitness activity using available mobile sensors, such as inertial measurement units (IMUs). Convolutional neural networks (CNNs) have been shown to produce strong results in different time series classification tasks, including the recognition of daily living activities. However, fitness activities can present unique challenges to the human activity recognition task (HAR), including greater similarity between individual activities and fewer available data for model training. In this paper, we evaluate the applicability of CNNs to the fitness activity recognition task (FAR) using IMU data and determine the impact of input data size and sensor count on performance. For this purpose, we adapted three existing CNN architectures to the FAR task and designed a fourth CNN variant, which we call the scaling fully convolutional network (Scaling-FCN). We designed a preprocessing pipeline and recorded a running exercise data set with 20 participants, in which we evaluated the respective recognition performances of the four networks, comparing them with three traditional machine learning (ML) methods commonly used in HAR. Although CNN architectures achieve at least 94% test accuracy in all scenarios, two traditional ML architectures surpass them in the default scenario, with support vector machines (SVMs) achieving 99.00 ± 0.34% test accuracy. The removal of all sensors except one foot sensor reduced the performance of traditional ML architectures but improved the performance of CNN architectures on our data set, with our Scaling-FCN reaching the highest accuracy of 99.86 ± 0.11% on the test set. Our results suggest that CNNs are generally well suited for fitness activity recognition, and noticeable performance improvements can be achieved if sensors are dropped selectively, although traditional ML architectures can still compete with or even surpass CNNs when favorable input data are utilized.

## 1. Introduction

Mobile fitness applications present a unique opportunity to provide users with real-time assistance during non-stationary fitness activities, such as athletics [1]. The first step towards such an application is the real-time recognition of different fitness activities using mobile sensor devices. Because commonly used approaches that rely on external video sensors such as [2] restrict the user to stationary activities, body-worn sensors, such as inertial measurement units (IMUs), must be used for a mobile application. Recognizing fitness activities based on IMU data is, in essence, a time series classification problem: given a sequence of sensor data points collected over a time period, predict the fitness activity performed during that time period.

Although most commonly used for visual tasks, convolutional neural networks (CNNs) have been shown to produce competitive results on time series classification tasks [3], including human activity recognition (HAR) [4,5,6]. In the context of fitness activity recognition, they have been successfully applied to various different activities, such as swing sports [7,8,9], skiing [10,11], beach volleyball [12], football [13], and exercising [14]. However, it is unclear how well CNN architectures translate to other fitness activities that can present unique challenges, such as low availability of training data, small differences between different activities, and limited processing power on mobile devices. Furthermore, most of these papers focus on their respective use cases and thus do not compare their CNN to other CNN architectures or traditional machine learning methods.

Therefore, this study aims to assess how CNN architectures can be adapted to the mobile fitness activity recognition task using IMUs and how their results compare to traditional machine learning. For this purpose, we propose a preprocessing pipeline, adaptations to three existing CNN architectures, and a new CNN architecture that aims to address the execution speed variability in fitness exercises. The performance of each architecture is evaluated on a running exercise data set that was recorded in the context of this study and compared to a baseline of three traditional machine learning models that are commonly used in HAR. Lastly, performance changes are determined for varying numbers of sensors and input data sizes.

### Contributions

Our work provides the following key contributions to the field of human activity recognition:

1. An introduction of the Scaling-FCN architecture designed for sensor-based fitness activity recognition with fixed time windows.
2. An introduction of a new public data set with IMU data of 20 participants for seven different running exercises [15].
3. A recording and preprocessing pipeline for fitness activity data recorded with multiple body-worn IMUs.
4. A detailed performance analysis of the Scaling-FCN compared to three existing CNN-based architectures and three traditional machine learning architectures on the running exercise data set, focusing on the effect of different input data parameters.

## 2. Data Acquisition

A representative data set is essential not only to train a machine learning model but also to assess its expected real-world performance. However, at the time of the study, we were unable to find a single public human activity recognition (HAR) data set that met the criteria for our study. In particular, we found that most data sets in the field of mobile HAR, such as the one provided by Anguita et al. [16] only cover activities of daily living and not fitness activities. Other data sets, such as the BasicMotions data set [17] and the CounterMovementJump data set [18], feature relatively few activities and only a single body-worn sensor. Furthermore, many public HAR data sets already consist of statistical features such as mean, minimum, and maximum values across a recording and, therefore, are not suitable for the CNN approaches evaluated in this study. The only data set that we could find that satisfies the previous criteria, the daily and sports activities data set by Barshan and Altun [19], consists of data from only eight subjects and primarily features activities that are very different from each other and, therefore, are relatively simple to classify. Plötz et al. [20] also acknowledge this lack of larger data sets in mobile HAR as one of its main challenges, appealing for the development of such data sets.

**Figure 1:**
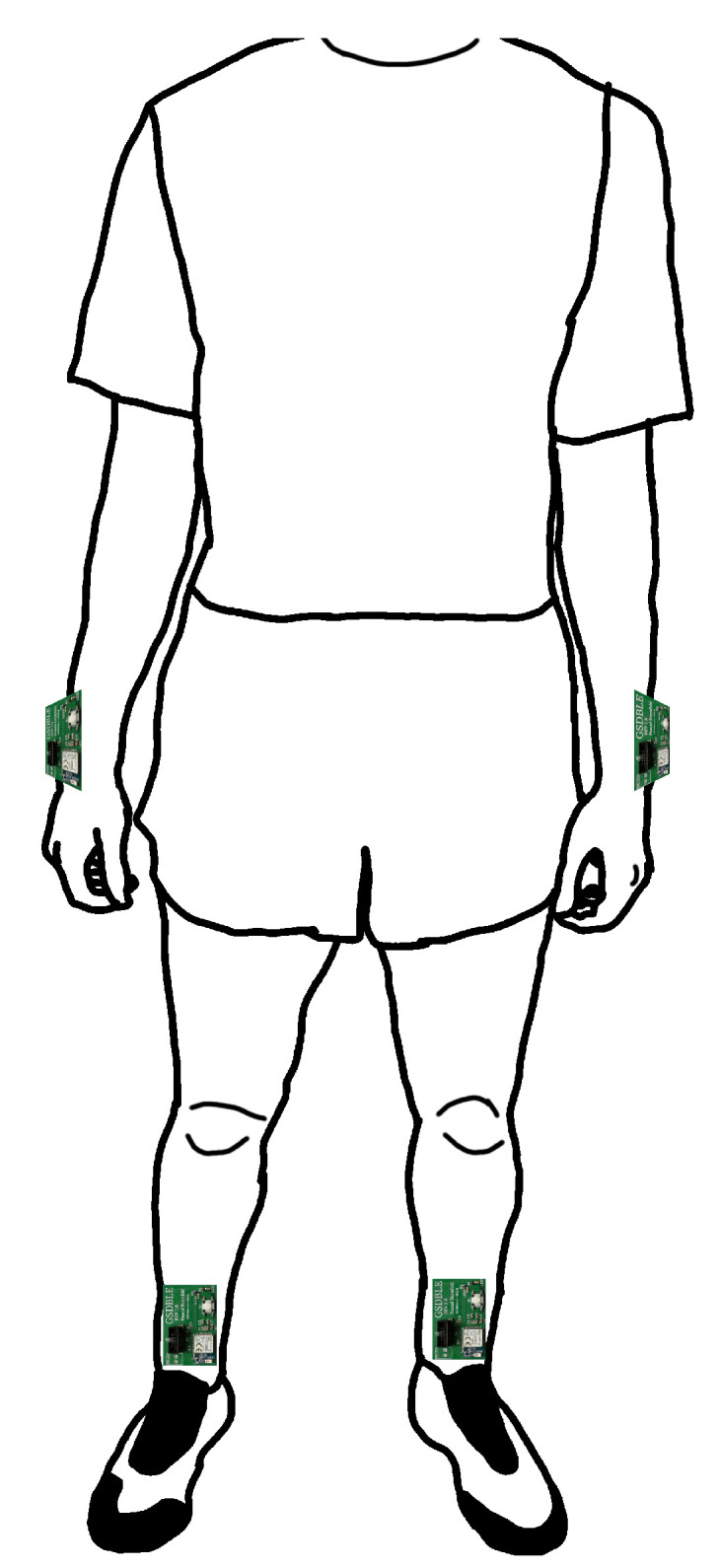
Position of the four GSDBLE sensor boards worn during data acquisition.

Therefore, we recorded a running exercise data set that is publicly available at [15]. The data set consists of seven popular running exercises performed by 20 healthy subjects (16 m, 4 f) between 16 and 31 years of age while wearing an IMU on each ankle and wrist for a total of four IMUs (see Figure 1).

### 2.1. Activity Choice

The fitness activities for our data set were chosen based on two primary criteria: subject availability and difficulty of classification. The availability of subjects, in particular, had to be taken into account because COVID-19 already limited the availability of subjects willing to participate in our study. We, therefore, could not afford to limit subjects to those active in specific sports. On the other hand, the different activities had to be sufficiently complex and similar to one another that classification would still prove challenging to classifiers. Based on these criteria, we chose the following running exercises that are performed as warm-up exercises in different types of sports:

- Regular running;
- Side skips (right and left direction);
- Carioca running (right and left direction);
- Heel-to-butt running;
- High-knee running;

Since we differentiate between two different directions for side skips and Carioca running each, we have a total of seven different fitness activity classes.

### 2.2. Hardware

**Figure 2:**
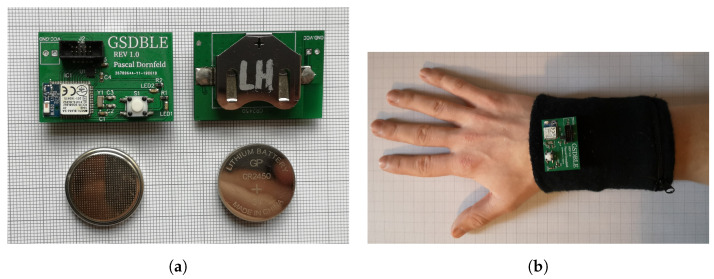
The data acquisition sensor setup. (**a**) GSDBLE sensor boards with coin batteries for scale. (**b**) The sweatband with the GSDBLE sensor board (currently not in pocket) on top.

Data were recorded using four GSDBLE sensor boards that have been developed for mobile activity recognition in the context of Pascal Dornfeld’s thesis [21] (see Figure 2a). They are powered by a CR2450 3 V lithium battery and record data with an LSM6DSL IMU from STMicroelectronics. They use a Bluetooth low energy (BLE) connection to send their accelerometer and gyroscope data alongside time stamps to a connected smartphone. The sensor boards are contained in sweatband pockets (see Figure 2b) so they can be worn without affecting the user’s mobility.

Subjects wore a total of four sensor boards during all recordings, one at each ankle and each wrist, respectively (see Figure 1). All sensor boards were connected to a single Huawei P20 smartphone that aggregated all their data using a custom recording application. At the end of a recording, the application stored the sensor data, timestamps, and metadata in a JavaScript Object Notation (JSON) format file. Timestamps are recorded in milliseconds, whereas accelerometer and gyroscope values are recorded as signed 16-bit integer values representing an acceleration of a∈[−16G,+16G] and angular velocity of v∈[−2000dps,+2000dps], respectively.

### 2.3. Output Data Rate & Data Loss

Since we could observe data loss with high sensor output data rates (ODRs) and four sensors connected to a single smartphone via BLE, we analyzed the incoming sensor data for data loss and timestamp inconsistencies. While our initial results suggested that data loss would only occur above 104 Hz, a 40 s real-world test performing fitness exercises already showed a significant increase in data loss when going from 52 Hz to 104 Hz (see Table 1). We, therefore, decided to use an ODR of 52 Hz when recording our data set since we expect the increased data rate to provide relatively little value when classifying human activities. Based on empirical tests with commercial devices that include an IMU, we expect the remaining data loss of roughly 3% to be representative of real-world applications when multiple devices are connected to a single device via BLE.

### 2.4. Recording Procedure

We recruited a total of 20 healthy young adults (16 m, 4 f, 16–31 yo) to participate in our study. All subjects stated that they engage in sport on a regular basis and know the presented or similar running exercises. Furthermore, each subject gave their informed consent to publish their anonymized recorded data. Each subject participated in one recording session, and one subject participated twice. During each recording session, a supervisor explained the scope of the study and ensured that no faulty data were recorded. In particular, they checked that the exercises were executed properly, that the sensors were worn properly, and that no hardware problems occurred. If such an issue was found during a recording, the recording was discarded and the last exercise was recorded again. Each exercise was recorded for 10 s per subject. The order of exercises was randomized for each subject individually to ensure that no data leakage could occur based on the order of exercise and the level of exhaustion of the subjects when performing each exercise. In practice, the order had to be slightly adjusted for some subjects to ensure that they could perform all the exercises. However, none of the subjects had to drop out of the study, resulting in complete data for all 20 subjects.

## 3. Preprocessing

To utilize the recorded data set in a machine learning (ML) classifier, it must be brought into a suitable format. In addition, recorded data should first be cleansed to reduce the impact of recording or transmission errors on classification performance.

### 3.1. Data Synchronization

**Figure 3:**
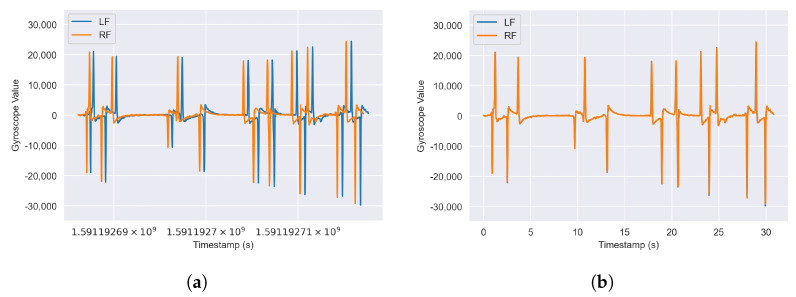
Comparison of sensor data streams for equivalent movements. (**a**) Before timestamp correction. (**b**) After timestamp correction.

When analyzing our data set, we found that the initial timestamps $t_{0}$ between different sensors of the same recording did not match exactly and drifted further apart over time (see Figure 3a). To address varying initial timestamps, we shift each sensor time series to start at t=0 by subtracting the respective sensor’s $t_{0}$ from all its timestamp values. To address the drift in timestamps over time, we determine a scaling factor $k_{n,r}$ for each sensor *n* and each recording *r* by dividing its last reported timestamp $t_{max}$ by the expected timestamp at the end of the recording $t_{exp}$. The timestamps of different sensors in each recording *r* are then scaled to match each other by dividing each timestamp by the respective scaling factor $k_{n,r}$ of its sensor *n*. In practice, we noticed that these scaling factors were constant between recordings for each sensor and thus determined a single global scaling factor $k_{n}$ for each sensor *n* that we applied to all its recordings. In a real-time application, this might necessitate a calibration process during which this scaling factor is determined for each connected sensor.

### 3.2. Processing Incomplete Data

Based on our findings in Section 2.3, we expect some data in a real-world setting to be lost during transmission. Whereas more elaborate approaches, such as local or global interpolation, exist to address missing data values, we opt for a duplication of the previous data value whenever an expected data value at a given timestamp is missing. In a real-time application, this allows for all data to be processed immediately instead of having to wait for the next data to interpolate a missing value. Based on our empirical findings, neither method provides a noticeable increase in classification performance over the other in our data set. However, a more elaborate approach might be preferable if a higher data loss is observed.

In the event that at least 10% of the expected samples were missing during the course of a 10 s recording for at least one sensor, the recording was completely discarded for the purpose of our evaluation. This was typically only the case when one sensor stopped sending data altogether and occurred four times in our 147 recordings, resulting in a total of 143 remaining recordings.

### 3.3. Standardization

Many machine learning architectures require the input data to be normalized or standardized for optimal training. Since we have two different types of data, acceleration and angular velocity, we use the standardization formula $V^{\prime }=\frac{V-\mu }{\sigma }$ to scale all data to μ=0 and σ=1 to prevent one type of data from dominating the other during training. The values for μ and σ were calculated once for all accelerometer data and once for all gyroscope data in the data set and then applied to all samples. We decided against calculating μ and σ for individual sensor positions and axes to preserve the relative differences in intensity. In a real-time application, these values could be supplied alongside the model to ensure consistent data values between training and inference.

### 3.4. Segmentation

**Figure 4:**
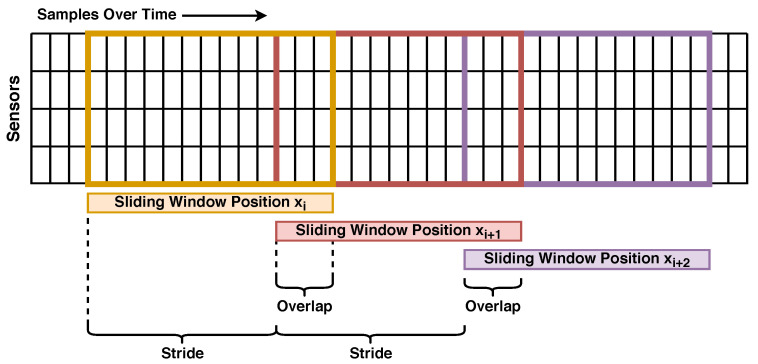
A visualization of the sliding window segmentation with a window size of 13, a stride of 10, and an overlap of roughly 23%.

To simulate the use case of a real-time application, data recordings have to be split into individual segments, each segment representing the data that last arrived at the application at a given point in time. Each segment later serves as one input sample in our experiments. For this purpose, we use a typical sliding-window approach, as shown in Figure 4.

Liu et al. [22] suggest that window length and overlap ratio are important parameters for HAR modeling and real-time performance and should be chosen with the data set and use case in mind, specifically mentioning the length of individual motions as useful a priori information, which they determined for daily activities in their previous work [23]. We estimate the length of individual running exercise repetitions to be roughly 0.5 to 1.0 s based on our observations that participants were able to perform roughly 10 to 20 repetitions in each 10 s recording. As a result, we chose a baseline window size of one second (52 timestamps) to ensure that each window contains at least one full repetition and compared it to smaller window sizes in Section 6.2.1, which may be better suited for real-time applications relying on low prediction latency, such as exergames. We use 75% as the overlap ratio to ensure that we have a sufficient number of samples for model training and to simulate a real-time application that requires regular prediction updates, resulting in a stride of 250 ms for a window length of one second. As detailed in the next paragraph, we strictly ensure that no data from the same user are shared between training, validation, and test sets, preventing any potential data leakage through window overlap.

When evaluating machine learning architectures, it is common practice to segment the given data set into three distinct partitions: a training set for model training, a validation set to assess model performance during design and optimization phases, and a test set to assess the final model performance on previously unseen data. To prevent data leakage between the different sets, we split our data set on a per-subject basis. This ensures that data from each subject is only included in a single set and that classes are represented equally in each set. It is representative of the typical real-world use case of a machine learning model having to predict a user’s activity without having seen data from the same user during training.

Our static test set consists of the data of four randomly selected participants, or 20% of all participants. For the training and validation sets, we instead use a leave-one-subject-out cross-validation approach to generate 16 different training/validation splits. By using the data of each participant not in the test set once for validation and fifteen times for training, we maximize the number of data in the training set while also minimizing the impact of individual participants’ data on validation results. As a result of this approach, each model is trained a total of 16 times for each scenario.

## 4. CNN Architectures

To account for the variety of CNN architectures available, we adapted three different CNN architectures that have been shown to perform well in time series classification tasks. Furthermore, we designed a fourth CNN architecture that utilizes data rescaling as proposed by Cui et al. [24] but adapted to the fitness activity recognition task.

Modifications to existing architectures were made when necessary to allow inference on mobile devices. Although recent work shows that current mobile phones are capable of running image classification model inference fast enough for real-time applications [25,26,27,28], and further optimizations are possible [29], implemented models should still aim to limit parameter count to preserve battery life and save computational resources. This can be particularly important in mobile HAR applications, where recognition models may run for prolonged periods of time in the background. Since we furthermore expect that the task of recognizing fitness activities will be less complex than the task of recognizing images, for which models usually contain at least one million parameters [25], we set one million as the upper limit for the parameter count of our models. In our empirical hyperparameter optimization, all architectures were able to generate optimal results (within the measurement error range) with between 207,360 and 302,400 parameters each, resulting in the final parameter counts shown in Table 2. All CNN architectures were implemented in PyTorch version 2.1 (https://pytorch.org/(accessed on 20 December 2023)).

### 4.1. Deep Convolutional Network (Deep-CNN)

Our Deep-CNN architecture is based on the VGG16 architecture introduced by Simoyan et al. [30], which has been used in successful image classification such as AlexNet [31] in the past. To reduce the size of the network for mobile real-time applications and adapt the network to smaller input sizes, we removed the first two blocks of convolutional layers, reduced the number of filters in the remaining three convolution blocks, and removed the pooling layers preceding each block of convolution layers. After each convolutional and fully connected layer, batch normalization was added. Lastly, we adjusted the size of the fully connected layers at the end of the model to fit the expected number of output classes and further limit the total parameter count. In total, the network has a total of nine convolution layers divided into three blocks, each doubling the number of filters in the previous block. ReLU was kept as the network’s activation function.

### 4.2. Fully Convolutional Network (FCN)

Instead of fully connected layers, fully convolutional networks (FCNs) use global pooling to generate inputs for the final softmax layer [32] and achieve impressive results in image segmentation tasks [33]. We use the FCN architecture of Wang et al. [34] as a baseline for our network. Since the architecture was already optimized for time series classification (TSC) and their model parameters are specified precisely, no large adjustments were necessary. As a result, we use the same architecture consisting of three convolution layers with 128, 256, and 128 filters, respectively, as well as an average pooling layer for global pooling. ReLU is again used as the activation function, whereas a softmax layer produces the final output.

### 4.3. Residual Network (ResNet)

Residual networks (ResNets) make use of residual connections to outperform regular CNNs with similar depths and parameter counts [35]. Wang et al. [34] again provide a ResNet that has been successfully applied in time-series classification and is used with minimal adjustments in our work. The model consists of three residual blocks with three convolution layers, followed by batch normalization and ReLU activation each. The final output is again generated using global pooling and a softmax layer.

Recently, modified and extended ResNet architectures specifically aimed at human activity recognition were proposed, including MAG-Res2Net [5] and an architecture [6] based on ResNeXt [36]. Whereas these architectures have shown promising results on the HAR data sets on which they were tested, the architecture proposed by Mekruksavanich et al. [6] does not compare ResNeXt with ResNet, and both publications were not available at the time of our study, leading us to use the regular ResNet architecture in this work. Given the strong performance of MAG-Res2Net on HAR data sets, it would be interesting for future work to assess their performance on fitness activity recognition data sets such as the running exercise data set proposed in this work.

### 4.4. Scaling-FCN

**Figure 5:**
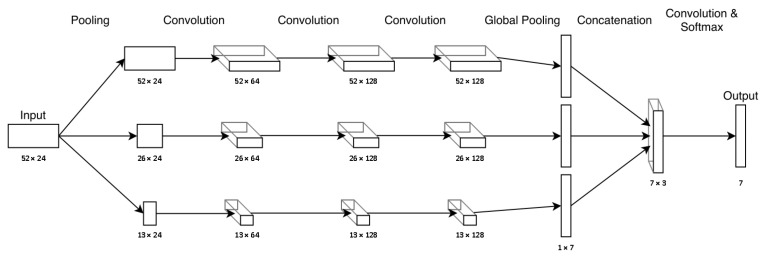
The Scaling-FCN architecture with data shapes for the Running Exercise data set with seven different classes.

The speed of execution of fitness activities can differ significantly between individuals based on factors such as fitness and motivation. Additionally, fitness activities often consist of multiple overlapping movements that may be performed at varying time intervals. We reflect this in our scaling fully convolutional network (Scaling-FN), shown in Figure 5, by using one-dimensional average pooling layers at the beginning of the network to rescale the input data in its time dimension. Compared to approaches such as the multiscale convolutional neural network of Cui et al. [24], our approach does not drop any data that could contain important information and is comparable to filtering in image classification, such as [37]. After scaling the input data to three different sizes using average pooling, each scaled input is processed in parallel by three convolution layers with a kernel size of 3 and a padding of 1, after which two-dimensional average pooling is applied. The data are then concatenated, fed into an additional convolution layer (*kernel_size* = 3, *stride* = 1, *padding* = 1), and finally fed into a softmax layer to generate the output. Similarly to ResNet, each convolution layer is followed by a batch normalization and a ReLU activation function.

## 5. Traditional Machine Learning

To create a baseline for CNN architectures, we used three traditional machine learning architectures: random forest (RF), support vector machine (SVM), and k-nearest neighbors (K-NN). We chose these particular architectures because they are commonly used in human activity recognition (HAR) and typically produce the best results among traditional machine learning approaches [38]. For each architecture, we use the respective default multiclass classifier implementation as found in scikit-learn version 1.3.0 (https://scikit-learn.org/stable/ (accessed on 13 November 2023)). As the support vector machine (SVM) and k-nearest neighbors (k-NN) architectures are not scale invariant, the feature data are first standardized as described in Section 3.3 to ensure optimal performance. Furthermore, all results are generated with a leave-one-subject-out cross-validation on the training set, as described in Section 3.4.

### 5.1. Features

For traditional machine learning algorithms, it is important to generate features that provide the model with the necessary information to differentiate classes. As surveys such as those conducted by Lara et al. [39] and Cornacchia et al. [40] show, a large number of different features in the time and frequency domains are being used for human activity recognition. Barandas et al. [41] propose the TSFEL library containing 60 different features for time series classification, which has been adapted by recent work in HAR, such as those by Rodrigues et al. [42], Liu et al. [43,44], and Hartmann et al. [45].

As we do not have the computational resources to perform an extensive feature selection procedure such as that detailed by Hui Liu [44] for multiple traditional machine learning architectures, we instead compare feature sets that each comprise all features of one category (statistical, temporal, and spectral) in TSFEL version 0.1.6 (https://github.com/fraunhoferportugal/tsfel (accessed on 8 December 2023)), respectively, in addition to all combinations of these feature sets. TSFEL currently supports 20 different statistical features, 14 different temporal features, and 26 different spectral features.

Table 3 shows the prediction accuracies for all combinations of feature sets for each of the three traditional machine learning architectures considered in this paper. For RF and AVM, a combination of statistical and temporal features achieves the best results, whereas for K-NN, this combination performs extremely close to the best-performing feature set consisting exclusively of temporal features. Furthermore, we could observe that the spectral features took significantly longer to generate than the statistical and temporal features. Table 4 shows the average combined time spent on feature generation plus prediction of a single label on a 4-core 8-thread 2200 Mhz Intel(R) Xeon(R) CPU. We expect these to be roughly representative of modern higher-end smartphone CPUs that have similar numbers of cores and frequencies, and we consider a prediction time of below 100 ms to be acceptable for real-time usage. As a result, we use the feature set that combines the 20 different statistical features and 14 different temporal features of the TSFEL library in all subsequent experiments. We opted against using a different feature set consisting of exclusively temporal features for K-NN as it only performed marginally better on the validation sets, performed worse on the test set (not shown here), and would have made it more difficult to compare the architectures’ behavior for different scenarios in Section 6.

### 5.2. Hyperparameters

A grid search was performed to determine the optimal hyperparameters for each architecture. A limit of 100 ms was set for the combined time of feature generation and prediction to ensure that the resulting models would still be suitable for a real-world use case. In practice, this limit did not have to be enforced, as none of the architectures ever surpassed it for any given hyperparameter combination. Table 5 shows the three architectures identified by their scikit-learn implementation and selected hyperparameter values for the hyperparameters that were optimized.

## 6. Results

The purpose of this study is to evaluate the usability of CNN architectures in the context of IMU-based recognition of fitness activities. For this purpose, we first determine a baseline using the traditional machine learning architectures presented in Section 5 that are commonly used for human activity recognition. We then compare the performance of these architectures with that of the CNN architectures presented in Section 4. Finally, we assess the performance impact when there are fewer sensor data available for classification.

For each scenario and architecture, we performed a leave-one-subject-out cross-validation with splits, as detailed in Section 3.4, resulting in 16 models each. CNNs were trained with a batch size of 128, early stopping after 15 consecutive epochs with no improvement, and a maximum of 1000 epochs. For each model trained during cross-validation, we additionally measured its performance on the test set that was not used during any training or hyperparameter optimization for any architecture. Therefore, we report performance metrics as the mean and standard deviation of the 16 trained models. As we have a well-balanced dataset, we will primarily present results using accuracy as a metric instead of resorting to less intuitive metrics such as the F1 score.

### 6.1. Architecture Performance

Table 6 shows the performance of all architectures during cross-validation and on the test set for the default input sensor data consisting of 1 s segments (52 sensor readings) with individual axis data for the four sensors (24 data per reading). For traditional machine learning, the features detailed in Section 5.1 were generated from the input data. It can be seen that RF and Deep-CNN generally perform worse than other architectures on the test and validation set, achieving scores between 94.54% and 95.57%. Of the remaining architectures, CNNs perform better on the validation set, reaching 98.37% in the case of FCN, while traditional architectures perform better on the test set, reaching 99.00% in the case of SVM. This suggests that the CNNs with their selected hyperparameters might be overfitting to the training and validation sets. Across all architectures, the standard deviation is significantly higher during cross-validation than on the test set, suggesting that the models perform significantly better for some people in the data set than for others. As seen in Table 7, CNN models generally stopped training well before the maximum of 1000 epochs was reached, suggesting that no potential performance was lost due to the maximum number of epochs. ResNet and Deep-CNN generally stopped training the earliest, with an average of 286 and 263 epochs, respectively, whereas our Scaling-FCN stopped the latest, with an average of 591 epochs.

**Figure 6:**
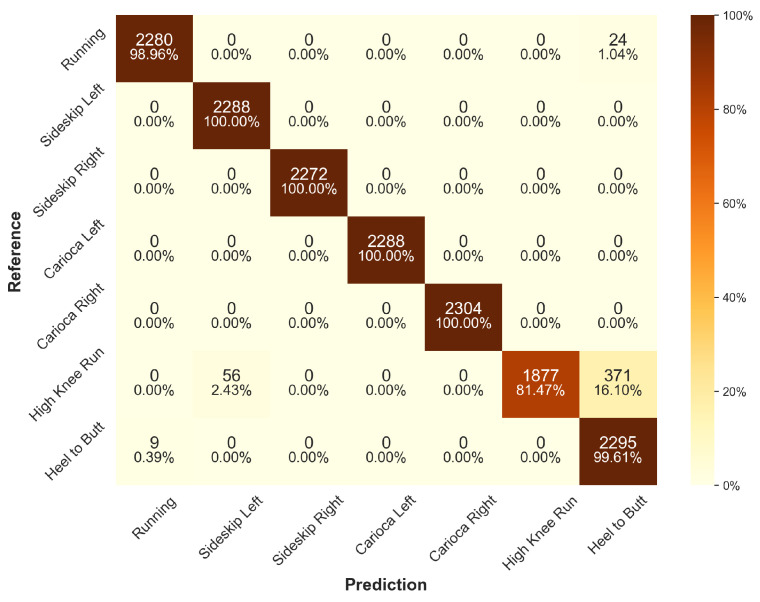
Confusion matrix for the ResNet architecture across all models of the cross-validation for the default scenario on the test data set.

**Figure A5:**
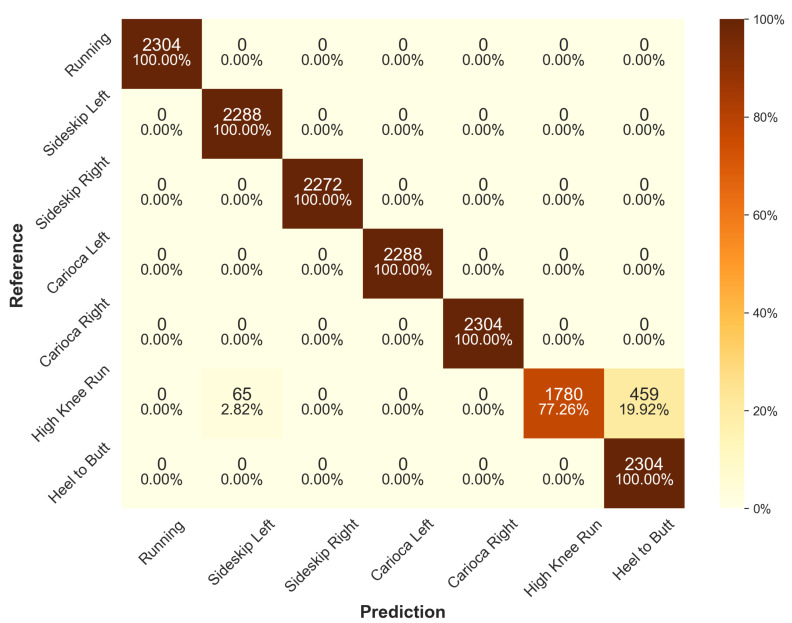
Confusion matrix for the FCN architecture across all models of the cross-validation for the default scenario on the test data set.

When looking at the confusion matrices of all trained models’ predictions on the test set (see Figure 6 for ResNet and Appendix A for other architectures), some common misclassifications can be observed. Most notably, all models share prediction errors for high-knee running, which is most commonly misclassified as heel-to-butt running, suggesting a similarity in the generated sensor data. Although the FCN (see Figure A5) has the highest misclassification rate for high-knee running, with only 77.26% accurately predicted, it is the only architecure with 100% prediction accuracy for all other classes. The second most commonly misclassified exercise is regular running, with up to 7.55% misclassifications in the case of Deep-CNN.

### 6.2. Impact of Sensor Data on Performance

To determine the impact of input sensor data on model performance, we repeated the previous experiment with varying time windows, sensor data dimensions, and sensor numbers. In the following, we present the most interesting findings and input data configurations with a focus on ResNet as the best-performing CNN on the test sets.

#### 6.2.1. Time Window Length

The time window length for the input data is an interesting parameter because it affects not only the total size of the input data but also the recency of the data used for classification. A reduced time window length might, therefore, be interesting when recognizing activity types where individual activities rapidly change, e.g., individual kicks and punches during kickboxing, because data from the previous activity are more quickly discarded. However, on our data set consisting of prolonged activities, we found that reducing the time window length always resulted in lower classification accuracy, as shown exemplarily for Resnet in Table 8 for window lengths of 52, 26, and 13 samples, corresponding to 1.0, 0.5, and 0.25 s of data each. Although there is a significant drop in accuracy when reducing the time window length, ResNet still achieves a respectable 94.96% accuracy with just 0.25 s of data, performing on par with or even outperforming traditional machine learning models with 1 s of data.

#### 6.2.2. Sensor Dimensions

By default, accelerometers and gyroscopes provide three-dimensional data, resulting in a total of six dimensions for each IMU. This may result in inconsistent data when the sensors are not applied exactly at the same location and in the same orientation. A potential solution is to instead use the rotation-invariant vector length of the vector spanned by the three dimensions of each sensor that represent total acceleration and total angular velocity, respectively. As seen in Table 9, ResNet still achieves a respectable 96.0% test accuracy with rotation-invariant data. Whereas the data show a performance gain when adding vector lengths as a fourth dimension to existing three-dimensional data, the difference is small enough to possibly be the result of variances between cross-validations.

#### 6.2.3. Sensor Number and Position

In a real-world scenario, wearing a sensor on each wrist and ankle may not be desirable or even possible. Therefore, we evaluated how CNN models perform for different subsets of our default sensor configuration. As can be seen in Table 10 for ResNet, the number and position of sensors can have a large impact on activity recognition. As expected for our running exercise data set, subsets without data from either foot decrease significantly in performance and only achieve approximately 83% test accuracy. Interestingly, model performance consistently improves when seemingly redundant data are removed, resulting in the highest accuracy of 98.59% being achieved when only data from a single ankle are used.

Although this behavior was consistent for all CNN models, traditional machine learning models did not share the same behavior and instead performed worse without data from either wrist. Table 11 shows the performance of all CNN and traditional machine learning models when only data from the right ankle are used. In this scenario, the test accuracies of traditional machine learning models dropped to between 89.80% for RF and 96.20% for SVM. With a test accuracy of 99.86%, our Scaling-FCN performs extremely well on this reduced problem, performing better than any other architecture in the process, although other CNNs also achieve scores of at least 98.44%.

Interestingly, all CNN models now perform worse during cross-validation than on the test set, suggesting that they may no longer overfit the training and validation sets. However, when we analyzed the individual models trained during cross-validation, it appeared that the data of a single participant could no longer be accurately classified for any sensor combination excluding the left foot, resulting in below 70% accuracy for that particular participant’s validation set for all architectures. Potential causes could be sensor movement relative to the foot or inconsistent execution of the movement pattern compared to other participants. Since neither was noticed during recording, we consider this representative of a real-world use case.

In an attempt to find the absolute best model on our data set, we also checked for combinations of the parameters assessed previously but could not find any combinations for which the CNNs performed better than for a time window of 52 samples and three-dimensional sensor data from a single ankle. In particular, all models performed worse with four-dimensional sensor data than they did with three-dimensional sensor data when only data from a single ankle were used.

### 6.3. Prediction Times

For a real-world use case, it is important to keep the prediction times and, thus, CPU usage low to preserve battery life and prevent the application from slowing down the smartphone. Furthermore, if the predictions are used as the input of real-time applications such as exergames, high prediction times directly result in high input latencies and, thus, a bad user experience. We recorded all models’ mean prediction times on the same 4 core 8 thread 2200 Mhz Intel(R) Xeon(R) desktop CPU, with CUDA disabled and no GPU attached. Table 12 shows the prediction times of all model architectures for the default scenario and the scenario where only data from the right ankle is used. For traditional machine learning architectures, times are reported as the sum of feature generation and prediction time, with pure prediction times in brackets.

Although all prediction times promise usability in a real-time application on a high-end smartphone, it is clear that the feature generation process presents a significant bottleneck for traditional machine learning models. Thus, a feature selection process that takes into account the feature generation time should be applied if real-time usage is desired. Furthermore, according to Hartmann et al. [45,46], a significant speedup by a factor up to the number of channels can be achieved by re-implementing the TSFEL features using NumPy vectorization. However, this was achieved on a 48-core desktop CPU and is unlikely to be representative of the speedup on a smartphone CPU. Nevertheless, efficient feature generation implementations, such as those suggested by Hartmann et al. [45,46], should be prioritized.

### 6.4. Limitations

Our results are primarily limited by the data set used in this work and by the specific model architectures evaluated. We expect the impact of the time window length and sensor positions, in particular, to be highly dependent on the activities in the data set. For example, slower weightlifting activities would likely benefit from longer input time windows and favor wrist sensor data over ankle sensor data. Therefore, while our results suggest that these parameters may be used to optimize performance, this behavior needs to be confirmed individually for future data sets.

Regarding the reference neural network architectures, we attempted to use established architectures with as few changes as possible to fit the requirements detailed in Section 4 to ensure comparability, but we cannot exclude that further hyperparameter tuning or architectural changes such as ResNeXt [36] produce better results than those presented in this work. For traditional machine learning architectures, we further cannot guarantee that the selected feature sets (see Section 5.1) are fully optimal, as our feature selection process was limited by the computational resources available to us.

Lastly, all prediction times were recorded on a 4-core 8-thread 2200 Mhz Intel(R) Xeon(R) desktop CPU. Real-world prediction times will highly depend on the respective smartphone CPU as well as background processes. Furthermore, our prediction times may benefit from a higher spatial and temporal locality of the accessed data compared to a real-world use case. As a result, our prediction times are primarily useful for a comparison between architectures and configurations and should not be taken as absolutes.

## 7. Conclusions

In this paper, we investigate the applicability of CNN-based architectures to the task of IMU-based fitness activity recognition. For this purpose, we designed a preprocessing pipeline, adapted three existing CNN architectures, and developed the Scaling-FCN architecture. Furthermore, we recorded a new data set [15] consisting of IMU data for seven different exercises performed by 20 participants, which is made publicly available. We evaluated the four CNN architectures by comparing their performance with three traditional machine learning architectures commonly used in human activity recognition and assessing the impact that different input data parameters had on their performance.

The results of our evaluation suggest that CNN-based architectures are well suited for IMU-based fitness activity recognition, consistently achieving strong results on our data set across a number of different input data configurations. Although K-NN and SVM outperform all CNNs on the test set when data from all four sensor positions are available, achieving up to 99.00% accuracy, FCN, ResNet, and our Scaling-FCN are still within at most three percentage points of K-NN and SVM on the test set while performing better on the validation sets. On our data set, CNNs show particularly strong performance when fewer input data are available, dropping by less than three percentage points when window sizes are reduced from 1.0 to 0.25 s. When only data from a single foot are available, CNNs outperform all traditional ML architectures on the test and validation sets, with our Scaling-FCN achieving up to 99.86% test accuracy.

In future work, we plan to investigate the performance of the Scaling-FCN within our pipeline on other data sets consisting of different fitness activities and ultimately apply it within the context of a mobile fitness application to track the user’s fitness activity and provide real-time feedback. As our data set is publicly available, we hope other scientists can utilize it to evaluate their systems and provide reference data for different machine learning architectures. Lastly, more research needs to be conducted to compare a larger variety of machine learning architectures on the task of fitness activity recognition on mobile devices, focusing on real-world applications.

## Figures and Tables

**Table 1:** Data loss (in %) during real-world usage performing fitness exercises over 40 s for each sensor and averaged across all sensors.

ODR	Data Loss Per Sensor (in %)	Average Data Loss (in %)
52	3.71 | 1.84 | 3.57 | 3.52	3.16
104	3.52 | 4.01 | 12.78 | 7.77	7.02

**Table 2:** Parameter count for each neural network architecture.

Architecture	Parameter Count
Deep-CNN	235,157
FCN	207,360
ResNet	302,400
Scaling-FCN	237,894

**Table 3:** Average model validation accuracy over 16 models on different feature sets.

Classifier	Feature Sets						
	**Stat.**	**Temp.**	**Spec.**	**Stat./Temp.**	**Stat./Spec.**	**Temp./Spec.**	**All**
RF	93.83%	89.72%	90.87%	94.34%	93.93%	90.87%	94.07%
SVM	96.84%	96.33%	91.82%	97.36%	94.45%	93.37%	95.2%
K-NN	91.64%	95.07%	89.18%	95.03%	92.54%	93.13%	94.05%

**Table 4:** Average prediction time including feature generation on different feature sets.

Classifier	Feature Sets						
	**Stat.**	**Temp.**	**Spec.**	**Stat./Temp.**	**Stat./Spec.**	**Temp./Spec.**	**All**
RF	67 ms	26 ms	306 ms	93 ms	373 ms	332 ms	400 ms
SVM	68 ms	27 ms	310 ms	95 ms	379 ms	337 ms	405 ms
K-NN	67 ms	26 ms	306 ms	94 ms	374 ms	333 ms	400 ms

**Table 5:** Traditional machine learning classifiers and selected hyperparameters.

Classifier	Hyperparameters
RandomForestClassifier	criterion = ‘entropy’ n_estimators = 400 max_depth = 10 min_samples_leaf = 1 min_samples_split = 4
SVC	C = 1 gamma = ‘scale’ kernel = ‘linear’
KNeighborsClassifier	n_neighbors = 12 p = 2 weights = ‘uniform’

**Table 6:** Average model accuracy over 16 models trained in a cross-validation.

Architecture	Test Accuracy (in %)	Validation Accuracy (in %)
RF	94.54 ± 0.94	94.99 ± 5.19
SVM	99.00 ± 0.34	97.64 ± 3.85
K-NN	98.55 ± 0.44	95.46 ± 5.06
Deep-CNN	95.57 ± 2.05	94.96 ± 6.36
FCN	96.74 ± 0.46	98.37 ± 4.91
ResNet	97.14 ± 1.36	97.89 ± 5.12
Scaling-FCN	96.46 ± 1.06	98.33 ± 5.04

**Table 7:** Average number of epochs for CNN model training.

Architecture	Epochs	Standard Deviation
Deep-CNN	286	112
FCN	439	138
ResNet	263	129
Scaling-FCN	591	270

**Table 8:** ResNet accuracy for varying time window lengths.

Window Length	Test Accuracy	Validation Accuracy
52	97.58 ± 1.36	97.57 ± 5.97
26	96.78 ± 0.89	96.68 ± 5.85
13	94.96 ± 1.21	96.35 ± 4.67

**Table 9:** ResNet accuracy for varying sensor dimensions.

Dimensions	Test Accuracy	Validation Accuracy
1	96.00 ± 1.39	97.24 ± 3.84
3	97.20 ± 1.04	97.40 ± 5.90
4	97.84 ± 1.41	97.68 ± 5.45

**Table 10:** ResNet accuracy for varying sensor position subsets.

Sensors	Test Accuracy	Validation Accuracy
All	97.02 ± 0.92	97.30 ± 5.88
Wrists only	82.71 ± 1.61	80.42 ± 11.26
Ankles only	96.37 ± 2.00	96.63 ± 6.88
Right side only	97.74 ± 0.72	99.75 ± 0.59
Right ankle only	98.59 ± 1.14	97.21 ± 9.56
Right wrist only	83.44 ± 2.21	84.22 ± 7.81

**Table 11:** Average model accuracy over 16 models trained in a cross-validation using only data from the right ankle.

Architecture	Test Accuracy (in %)	Validation Accuracy (in %)
RF	89.80 ± 1.18	92.03 ± 6.48
SVM	96.20 ± 0.78	96.30 ± 4.03
K-NN	94.55 ± 0.44	93.39 ± 5.00
Deep-CNN	98.44 ± 0.98	96.47 ± 7.92
FCN	99.22 ± 0.56	97.23 ± 7.88
ResNet	98.60 ± 1.16	97.25 ± 7.98
Scaling-FCN	99.86 ± 0.11	97.11 ± 9.85

**Table 12:** Average prediction time of each architecture on the test set, including feature generation for traditional architectures. Pure prediction times are reported in brackets.

Architecture	All Sensors	Right Ankle
RF	93.50 ms (0.08 ms)	23.42 ms (0.07 ms)
SVM	93.89 ms (0.47 ms)	23.44 ms (0.09 ms)
K-NN	93.69 ms (0.27 ms)	23.47 ms (0.11 ms)
Deep-CNN	0.89 ms	0.88 ms
FCN	1.59 ms	1.66 ms
ResNet	1.67 ms	1.72 ms
Scaling-FCN	1.98 ms	1.71 ms

**Figure A1:**
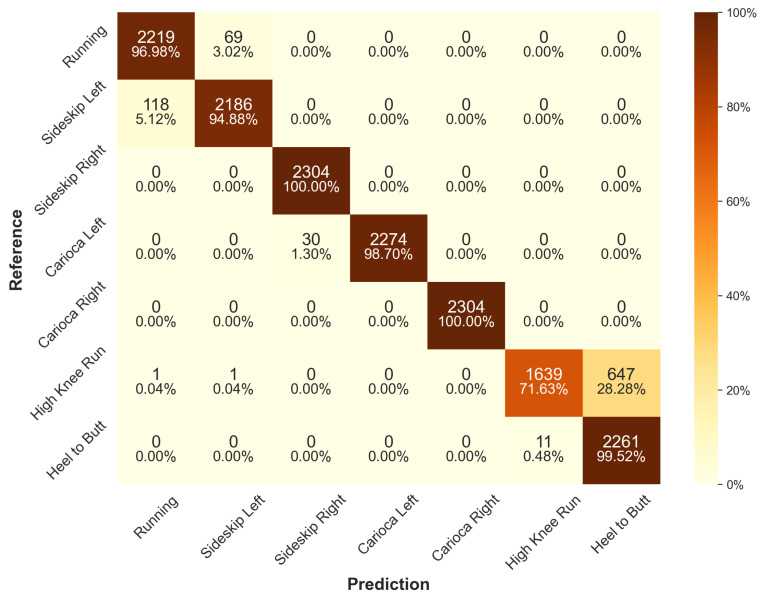
Confusion matrix for the RF architecture across all models of the cross-validation for the default scenario on the test data set.

**Figure A2:**
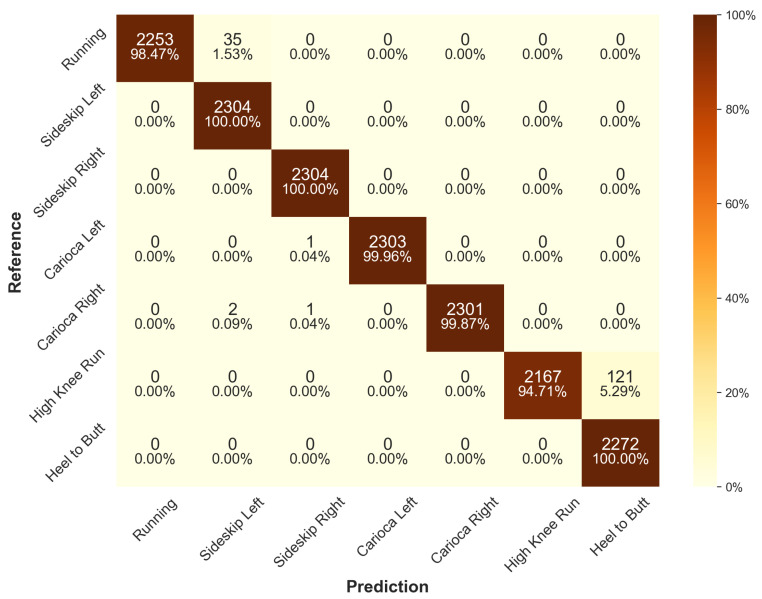
Confusion matrix for the SVM architecture across all models of the cross-validation for the default scenario on the test data set.

**Figure A3:**
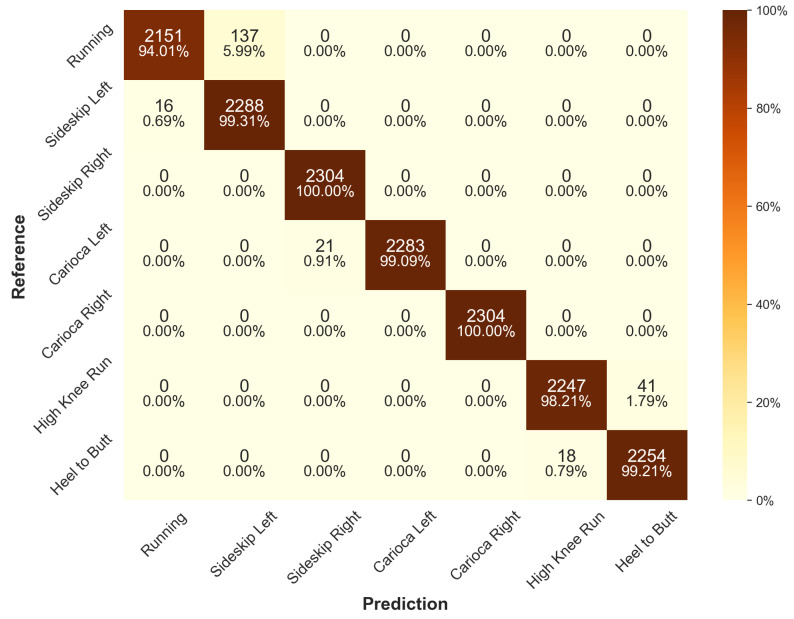
Confusion matrix for the K-NN architecture across all models of the cross-validation for the default scenario on the test data set.

**Figure A4:**
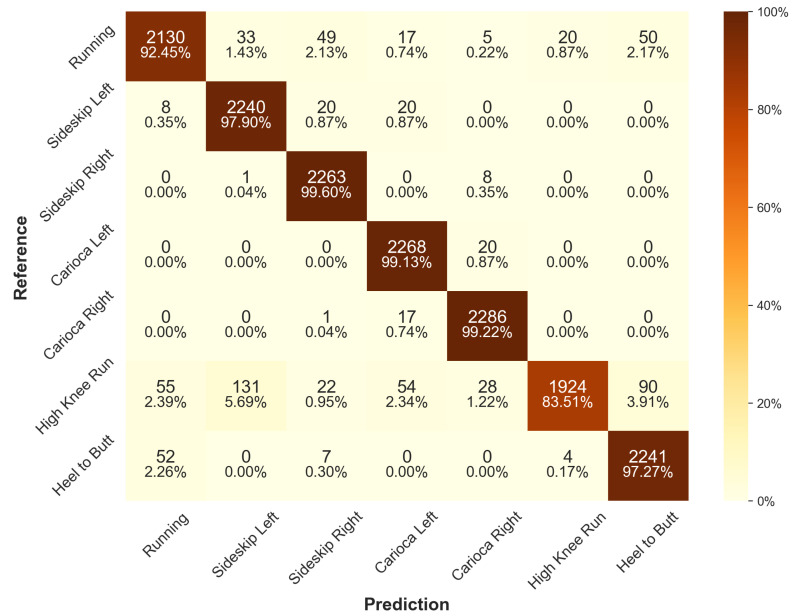
Confusion matrix for the Deep-CNN architecture across all models of the cross-validation for the default scenario on the test data set.

**Figure A6:**
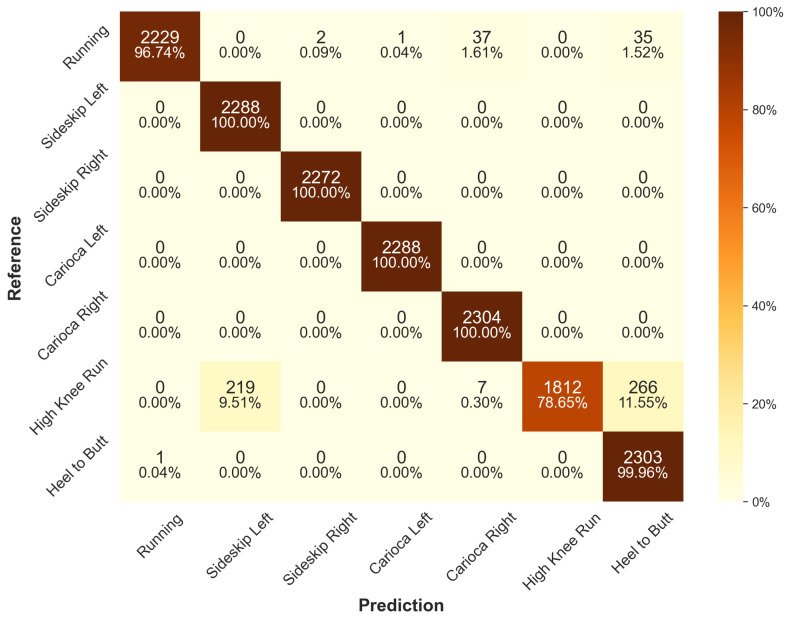
Confusion matrix for the Scaling-FCN architecture across all models of the cross-validation for the default scenario on the test data set.

## Data Availability

The data set is available at https://figshare.com/articles/dataset/Running_Exercise_IMU_dataset/22117235 (accessed on 17 Feburary 2023).

## Abbreviations

The following abbreviations are used in this manuscript:

AR	augmented reality
TSC	time series classification
HAR	human activity recognition
FAR	fitness activity recognition
ML	machine learning
DL	deep learning
ANN	artificial neural network
CNN	convolutional neural network
ResNet	residual neural network
Deep-CNN	deep convolutional neural network
FCN	fully convolutional network
Scaling-FCN	scaling fully convolutional network
RF	random forest
SVM	support vector machine
K-NN	k-nearest neighbor
IMU	inertial measurement unit
BLE	Bluetooth low energy
ODR	output data rate
JSON	javascript object notation

## Appendix A. Confusion Matrices

The following confusion matrices show the per-class performance of individual architectures’ models trained during cross-validation for the default scenario detailed in Section 6.1.
